# Generalizability of deep learning models for dental image analysis

**DOI:** 10.1038/s41598-021-85454-5

**Published:** 2021-03-17

**Authors:** Joachim Krois, Anselmo Garcia Cantu, Akhilanand Chaurasia, Ranjitkumar Patil, Prabhat Kumar Chaudhari, Robert Gaudin, Sascha Gehrung, Falk Schwendicke

**Affiliations:** 1 Department of Oral Diagnostics, Digital Health and Health Services Research, Charité - Universitätsmedizin Berlin, Aßmannshauser Str. 4-6, 14197 Berlin, Germany; 2 Department of Oral Medicine and Radiology, King George’s Medical University, Lucknow, India; 3 Division of Orthodontics and Dentofacial Deformities, AIIMS, New Delhi, India; 4 Department of Oral and Maxillofacial Surgery, Charité - Universitätsmedizin Berlin, Berlin, Germany

**Keywords:** Dental diseases, Computer science

## Abstract

We assessed the generalizability of deep learning models and how to improve it. Our exemplary use-case was the detection of apical lesions on panoramic radiographs. We employed two datasets of panoramic radiographs from two centers, one in Germany (Charité, Berlin, n = 650) and one in India (KGMU, Lucknow, n = 650): First, U-Net type models were trained on images from Charité (n = 500) and assessed on test sets from Charité and KGMU (each n = 150). Second, the relevance of image characteristics was explored using pixel-value transformations, aligning the image characteristics in the datasets. Third, cross-center training effects on generalizability were evaluated by stepwise replacing Charite with KGMU images. Last, we assessed the impact of the dental status (presence of root-canal fillings or restorations). Models trained only on Charité images showed a (mean ± SD) F1-score of 54.1 ± 0.8% on Charité and 32.7 ± 0.8% on KGMU data (*p* < 0.001/t-test). Alignment of image data characteristics between the centers did not improve generalizability. However, by gradually increasing the fraction of KGMU images in the training set (from 0 to 100%) the F1-score on KGMU images improved (46.1 ± 0.9%) at a moderate decrease on Charité images (50.9 ± 0.9%, *p* < 0.01). Model performance was good on KGMU images showing root-canal fillings and/or restorations, but much lower on KGMU images without root-canal fillings and/or restorations. Our deep learning models were not generalizable across centers. Cross-center training improved generalizability. Noteworthy, the dental status, but not image characteristics were relevant. Understanding the reasons behind limits in generalizability helps to mitigate generalizability problems.

## Introduction

In recent years, the analysis of medical images in a range of disciplines, e.g. dermatology, ophthalmology and radiology has been increasingly assisted by the application of multi-layered (deep) neural networks, a technique known as deep learning^1^. In dentistry, deep learning has been successfully applied to detect caries on peri-apical and bitewing images, as well as periodontal bone loss and apical lesions on panoramics and peri-apicals^2^.

Deep neural networks learn representations of statistical patterns and inherent structures from a large amount of data. In particular, deep convolutional neural networks (CNN) are suited to abstract highly complex spatial patterns from images. These models are trained in a supervised manner, by repeatedly presenting data points (e.g. images) and their corresponding labels (e.g. “apical lesion present”). Along this learning process, the internal parameters (weights) of the CNN are iteratively adjusted by minimizing a loss function, i.e. a quantifier of the deviation of the model predictions from the known labels^1^.

A range of limitations in deep learning applications in medicine have been identified^3^. Among those is the uncertainty about the generalizability of the developed models, i.e. their capacity to adequately predict on data which sources differ from those involved in the model training^4^. Hence, the use of independent datasets for model evaluation is recommended, as deep learning models trained and evaluated in-sample are at the risk of being over-parametrized, i.e. of “memorizing” the data. Under such conditions, the evaluation of the model may result in overly optimistic assumptions about its overall performance^5,6^. Limited generalizability of deep learning models may be related to differences in image characteristics (associated with different data generation protocols, e.g. machine types or acquirement settings) or population characteristics (e.g. age, sex, dental status etc.)^5^.

The generalizability of deep CNNs in medicine has not been widely evaluated, and there is currently no study available on this matter in dentistry. Moreover, elucidating the causes underlying possible deficits of generalizability is relevant, as this can facilitate the development of improved modeling strategies to overcome this problem as well as to define standards for model benchmarking prior to clinical usage to ensure robustness and generalizability. In the present study, we assessed the generalizability of deep CNNs for detecting apical lesions on panoramic radiographs. Our hypothesis was that a model developed on data from only one population, characterized by image and cohort features, shows significantly worse performance on unseen imagery from another population. Beyond gauging the models’ generalizability, our analysis focused on investigating the causes of limited generalizability and consequently on possible improvements in model training strategies.

## Materials and methods

### Study design

This study employed two datasets of panoramic radiographs from two centers, one in Germany (Charité, Berlin) and one in India (King George Medical University, Lucknow, KGMU). Images had been pixel-wise annotated for apical lesions by four independent dental specialists and a master reviewer. U-Net type deep CNNs were trained to detect apical lesions. The models were trained using different proportions of data from the two centers. A range of experiments was performed to explore possible sources of differences in model performance when evaluating them on different datasets (with different proportions of data from both centers). Reporting of this study follows the STARD guideline^7^ and the Checklist for Artificial Intelligence in Medical Imaging, CLAIM^8^.

### Performance metrics

Model performance was assessed using a binary classification of every single pixel contained in an image, employing four performance metrics; F1-score, sensitivity, predicted positive value (PPV), and specificity. During training, the model was validated using the mean intersection-over-union score. Details on the metrics are provided in the appendix.

### Sample size

Our primary outcome metric was the F1-score, which was suitable to reflect on the imbalances in our dataset (only a minority of pixels of any image are associated with apical lesions). For sample size estimation, we considered an independent two-sided t-test to compare the F1-score of the same models on Charité test data (assumed to be F1 = 50%) versus KGMU data (F1 = 45%), and conservatively assumed a standard deviation of 15% in both groups, a power of 1-beta = 0.80 and alpha = 0.05. Under these assumptions, we required a minimum of 143 images per group. The final test set consisted of 150 images per group.

### Image dataset

The analysis involved a set of 1300 panoramic radiographs, each of them cropped in order to isolate the region of interest, as shown in the appendix^9,10^. A total of 650 images were provided by Charité and 650 images by KGMU, respectively. Only radiographs from dentate adults were included, without any additional image selection criteria. The collection of data was ethically approved (EA4/080/18). Charité images were generated by radiographic devices from Sirona Densply (Bensheim, Germany) and Dürr Dental (Bietigheim-Bissingen, Germany), while KGMU images were produced using Planmeca machines (Helsinki, Finland), both at different tube voltages and exposure times (depending on age and sex of the patient, among other parameters).

### Reference set

The panoramic images were labeled pixel-wise by dental experts, each with at least 4 years of experience. Each annotator independently assessed each image under standardized conditions using an in-house custom-built annotation tool as described before^11^. Prior to annotation, the examiners were advised on how to discriminate apical lesions from other entities (e.g. endodontic-periodontal lesions, a widened periodontal ligament etc.), as described in detail elsewhere^12^. Each image was independently assessed by four experts. In a second step, the provided annotations were reviewed (addition, deletion, confirmation) by another expert with at least 10 years of experience and a focus on conservative dentistry and endodontology. Finally, the reference set was established as the union of all pixel labels on each image.

### Data preparation, model and training

A fully convolutional neural network of the U-Net type^13^ was trained to detect apical lesions. The model performs segmentation of images, i.e. classifies each pixel in an input image and thereby explicitly reproduces the spatial coverage of the object of interest (here, apical lesions). The U-Net architecture consists of encoding and decoding parts. The encoder abstracts image features that and the decoder uses this information to reconstruct the spatial coverage. The EfficientNet-B5 encoder was used, with its weights being initialized with those of a network previously trained to detect caries lesions on bitewings^11^. The optimization of the model weights themselves was based on the backpropagation algorithm and the binary focal loss function, which is a generalization of the binary cross-entropy loss.

The initial training set consisted of 500 images, all of them containing at least one apical lesion (= positive annotations) selected at random from the Charité dataset. The decision was taken to reduce class imbalance on pixel level to some degree (the majority of pixels will nevertheless be negative, i.e. not affected by apical lesions). Prior to training, all images were re-scaled (width = 704 px, height = 352 px). For training, a fivefold cross-validation approach was used (see Fig. A1a in appendix). To ensure similarity of these cross-validation splits, each dataset (Charité, KGMU) was subset into clusters of homogenous dental status with respect to the number of apical lesions per image and the number of posterior and anterior teeth. To generate these clusters, a mini batch K-means clustering algorithm^14^ was used, with the optimal number of clusters being established by quantifying the Silhouette^15^ and the Davies–Bouldin^16^ scores. Clustering generated two data subsets, one featuring high and the other low number of teeth. In both sets the mean number of apical lesions per image was similar. Images from both clusters were eventually combined in order to achieve homogeneous cross-validation splits.

**Figure 1:**
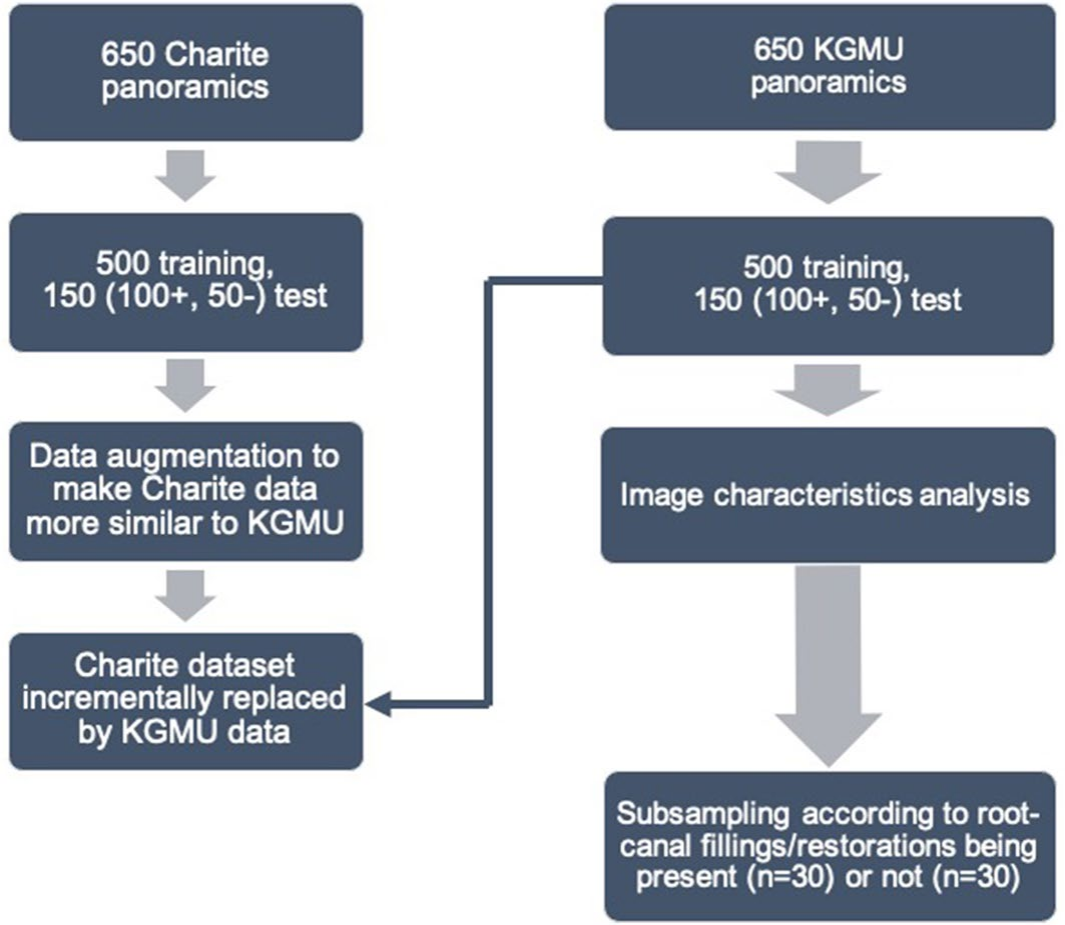
Flowchart of the experimental workflow. From both centers, 650 panoramic images were used to train and validate (500 images per center) and test (150 images per center, 100 with apical lesions, 50 without, respectively) models. Image characteristics were compared, the Charité training dataset augmented accordingly and then the re-trained model was tested on KGMU data. Further, models were trained on an increasingly mixed Charité-KGMU dataset and tested on KGMU data. Last, models were tested on subsamples of KGMU data consisting root-canal fillings (and other restorations) or no root-canal fillings/restorations at all.

U-net models were trained and validated for every train-validation split, thus yielding 5 different models. During training, the images were augmented by applying random geometric transformations. The models were optimized by minimizing a linear combination of binary cross entropy and Dice loss. The learning rate was set to 0.002 and the batch size to 4. The output of each model was binarized by selecting a cutoff optimizing the F1-score. The training was stopped after 200 epochs. Convergence was assessed by monitoring the mean IoU on the validation set. After every epoch the model was evaluated on the validation split. The best performing model evaluated on each of the validation splits was selected. Each of the finally selected five models was evaluated on the test set (see below) and their metrics average reported (see Fig. A2b in the appendix). Model training and data augmentation were carried out on a GeForce GTX 1080 Ti GPU, using Keras and the imgaug library.

**Figure 2:**
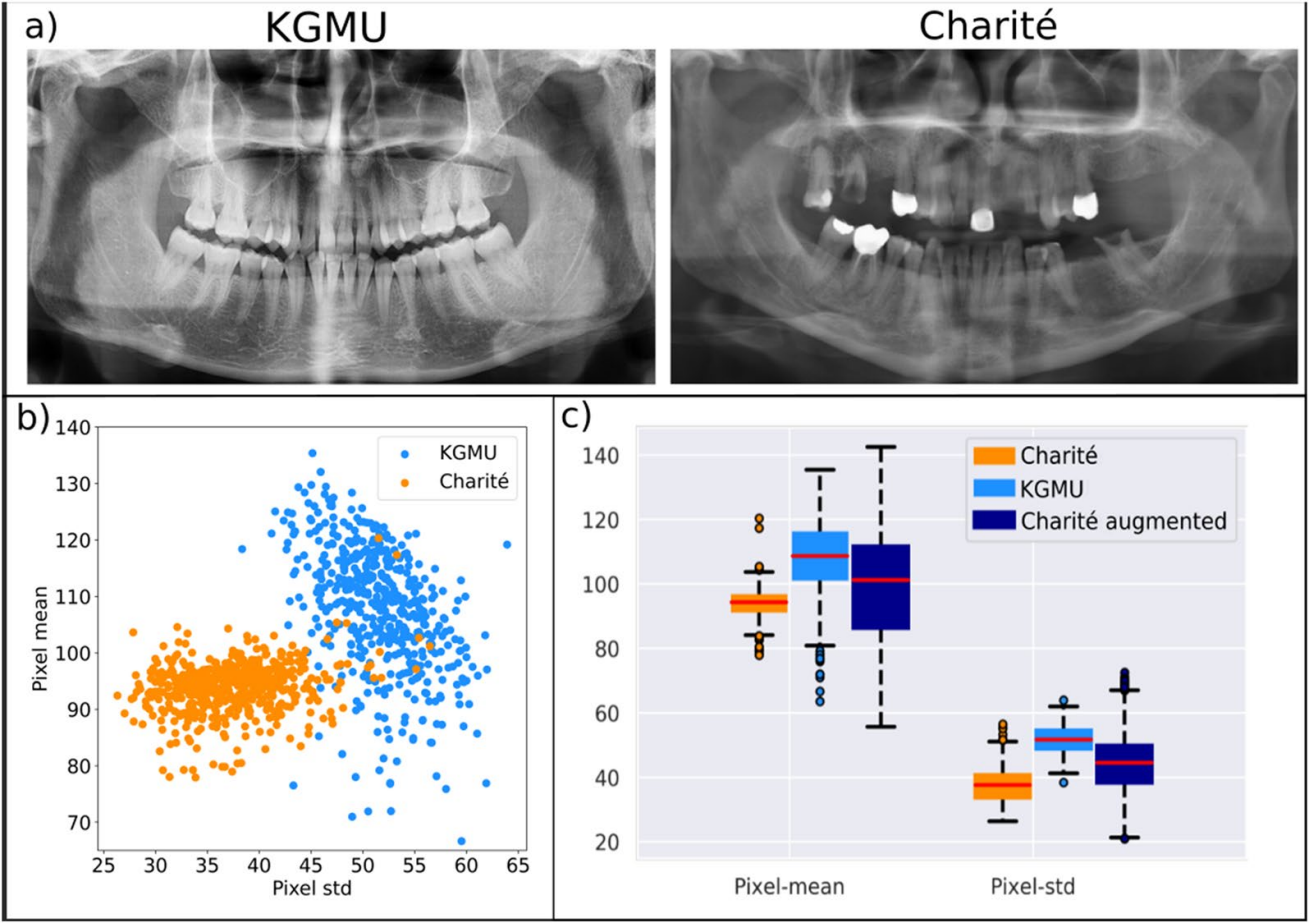
Differences between the Charité and KGMU sets. (**a**) Representative images of the KGMU and Charité datasets, showing the typical observed differences in population characteristics (number of teeth, presence of dental restorations) and image conditions (brightness and contrast). (**b**) The plot of the pixel means (proxy for brightness) and standard deviations (proxy for contrast) of images included in the training sets. (**c**) Boxplots of the pixel means and standard deviations of the Charité and KGMU images in the training sets as well as of the same Charité images after application of the pixel-wise transformations by data augmentation. Box and line: 25/75th percentiles and median. Whiskers: Minimum/maximum or 1.5 inter quartile rage if outliers are present; Dots: Outliers.

### Testing generalizability

Two main test sets (Charité, KGMU) were constructed, each consisting 150 images (100 with positive annotations and 50 with negative ones, i.e. without apical lesions). Starting with a training set containing only radiographs from Charité (see above), the trained model was tested on both these test sets, respectively, to gauge generalizability. In a second step, the relevance of image characteristics for generalizability was explored. To do so, pixel value transformations were included in the data augmentation, thus aligning the two data sets with respect to pixel value distributions, shifting the mean and standard deviation of the pixel values of Charité images towards the mean and standard deviation of pixels values of KGMU. This was done by modifying the brightness and contrast of the images by random factor multiplication and by applying Contrast Limited Adaptive Histogram Equalization, respectively. Notably, these pixel augmentations were executed in absence of geometric transformations as these can artificially introduce black pixels at the boarders of the images. In a third step we evaluated cross-center training effects on generalizability, i.e. how introducing KGMU images to the initial training and validation dataset (incrementally and randomly replacing images from Charité) impacts on the models’ generalizability. Replacement was performed to hold the overall dataset size constant. Last, we assessed the impact of the dental status, here characterized by the presence of root-canal fillings or restorations (fillings, crowns, bridges), on generalizability. Therefore, two subsamples (each of 30 positive images) were sampled from the KGMU test set, one where images lacked root-canal fillings or restorations and the other where each image contained root-canal fillings and restorations.

### Statistical analysis

Differences in model performance were evaluated via independent two-sided t-tests, using *p* < 0.05 as discriminating criterion. Computations were performed using the Python library scipy 1.5.2^17^.

### Ethical approval and informed consent

All experiments were carried out in accordance with relevant guidelines and regulations. Data collection was ethically approved (Charité ethics committee EA4/080/18).

## Results

### Dataset characteristics

The KGMU and Charité datasets showed differences in the population characteristics (including dental status) as well as in image characteristics (Table 1). Patients in Charité showed fewer teeth in both the anterior and posterior area, but more restorations (fillings, crowns) and root-canal fillings per image than in KGMU. On the other hand, KGMU images were brighter and exhibited higher contrast. Details on the clusters used to generate homogenous cross-validation splits are also shown in Table 1.

**Table 1:** Characteristics of the different datasets.

Patient/Image features	Charité				KGMU					
	Train/validation			Test	Train/validation			Test		
	Full set	Cluster low	Cluster high		Full set	Cluster low	Cluster high	Full set	With restorations	Without restorations
No. anterior teeth; Mean (std, min–max)	10.53 (2.68, 1–12)	8.51, (3.37, 2–12)	11.7, (0.95, 1–12)	10.54, (2.89, 1–12)	10.92, (2.26, 0–12)	8.38, (3.55, 0–12)	11.51, (1.25, 2–12)	1.60, (1.23, 2–12)	11.9, (0.54, 9–12)	11.3, (1.99, 2–12)
No. posterior teeth; Mean (std, min–max)	12.03, (5.32, 0–20)	6.09, (2.88, 0–10)	15.5, (2.67, 11–20)	12.90, (5.47, 0–20)	16.62, (3.79, 1–20)	9.97, (3.0, 1–16)	18.18, (1.78, 14–20)	17.48, (2.86, 6–20)	17.43, (2.68, 7–20)	17.34, (3.0, 6–20)
No. apical lesions; Mean (std, min–max)	2.10, (1.5, 1–13)	2.08, (1.5, 1–13)	2.12, (1.5, 1–8)	1.6, (2.25, 0–16)	2.12, (1.46, 1–8)	2.20, (1.28, 1–5)	2.11, (1.49, 1–8)	1.25, (1.24, 0–6)	1.77, (0.99, 1–5)	1.57, (0.92, 1–5)
Images with fillings (%)	0.87	0.73	0.94	0.61	0.32	0.20	0.34	0.34	0.83	0
Images with crowns and/or bridges (%)	0.76	0.8	0.75	0.62	0.17	0.20	0.16	0.13	0.37	0
Images with root-canal fillings (%)	0.78	0.7	0.82	0.82	0.28	0.27	0.29	0.28	1.0	0
Median image pixel-mean (Quartile 1, Quartile 3)	94.27 (91.1, 96.7)	–	–	–	–	–	–	108.67 (101.0, 116.3)	–	–
Median image pixel-std (Quartile 1, Quartile 3)	37.55 (33.06, 41.30)	–	–	–	–	–	–	51.68, (48.38, 55.21)	–	–

For the Charité and KGMU training sets, the clusters featuring a high/low number of teeth, as well as the full set where both clusters were combined. In the case of the KGMU test set, two additional subsamples were considered, one with root-canal fillings and restorations being present and the other without any.

### Model performance and generalizability

The experimental and data flow is summarized in Fig. 1. In the first experiment, models trained only on Charité images showed a (mean ± SD) F1-score of 54.1 ± 0.8% if evaluated on Charité test data and 32.7 ± 0.8% on KGMU test data, respectively (*p* < 0.001, t-test). The limited generalizability was mainly grounded in a lower sensitivity on KGMU versus Charité data (48.0 ± 1.0% on Charité vs 22.0 ± 1.3% on KGMU, *p* < 0.001). Only limited and non-significant differences between both data sets were observed for the PPV (64.0 ± 4.0% Charité vs 63.0 ± 3.0% KGMU) and specificity (99.95 ± 0.01% Charité vs 99.97 ± 0.01% KGMU).

In a second experiment pixelwise augmentation related to the brightness and contrast was applied to the Charité training set, aligning the distributions of the mean and standard deviation of the image pixels towards KGMU image characteristics (Fig. 2). We found that training the models on images sharing similar pixel values did not lead to significant differences in F1-scores and did not improve generalizability. Moreover, this augmentation significantly lowered the sensitivity on both Charité (45.7 ± 1.2%, *p* < 0.02) and KGMU (19.2 ± 1.6%, *p* < 0.02) images.

**Figure 3:**
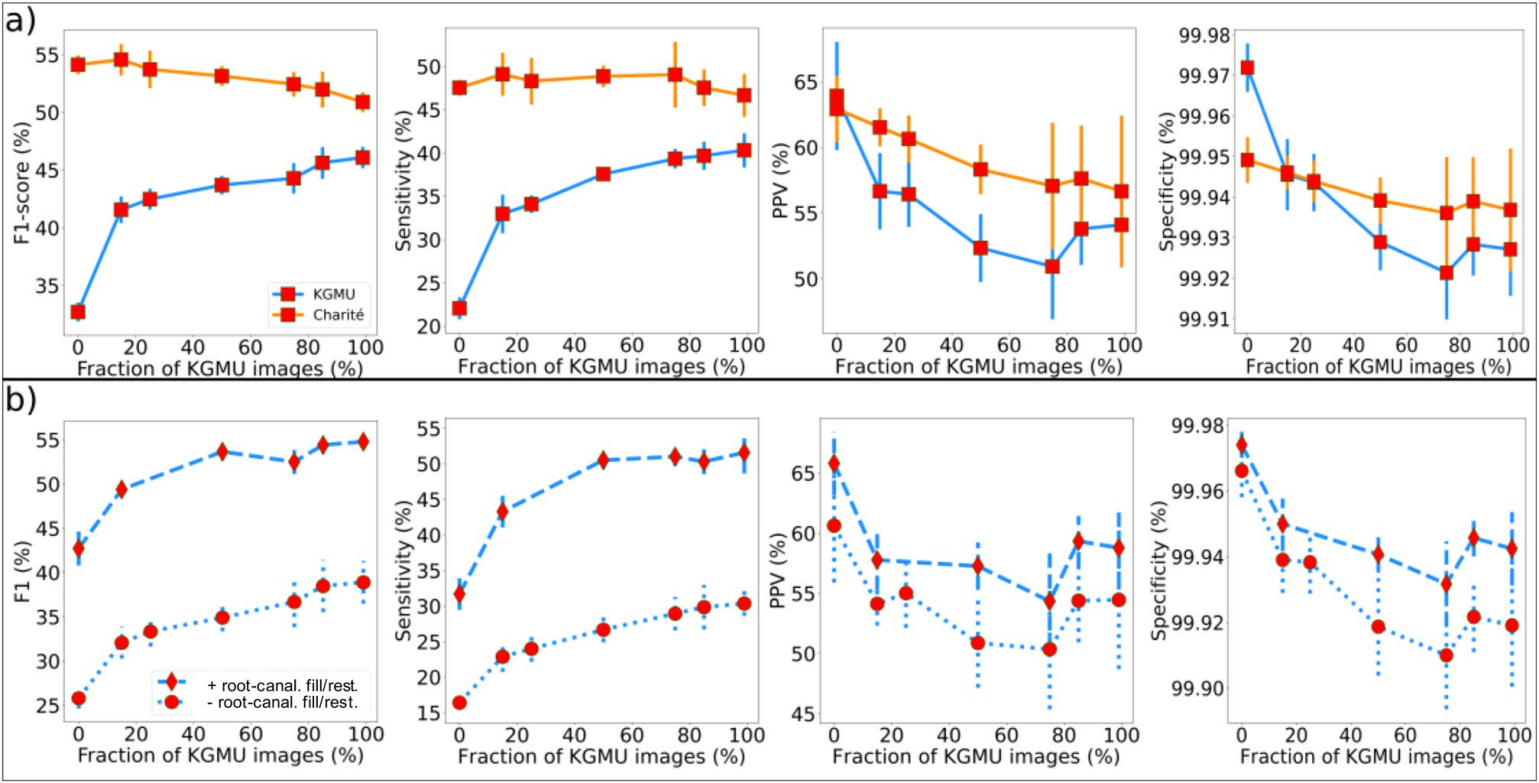
(**a**) The model performance on the Charité and KGMU test sets with different fractions of KGMU images in the training set (markers and error bars denote the mean and standard deviation of the scores over the set of the 5 best models selected from cross-validation, respectively). (**b**) Model performance for the subsamples of the KGMU dataset with and without root-canal fillings and restorations being present. *PPV* Positive predictive value.

In a third experiment we assessed the impact of gradually increasing the fraction of KGMU images in the training set. Increasing this fraction from 0 to 100% had the F1-score on KGMU images increasing monotonically to reach 46.1 ± 0.9 (Fig. 3a) as well as sensitivity (40.3 ± 2.0%, *p* < 0.001). On the other hand, a decrease was observed for PPV (to 54.1 ± 3.2%, *p* < 0.01) but not specificity (99.93 ± 0.02%; *p* > 0.05). Concomitantly, the increase of KGMU data reduced the F1-score on Charité images (to 50.9 ± 0.9%, *p* < 0.01) as well as the PPV (56.66 ± 5.75%, *p* < 0.01), without significantly modifying the sensitivity or specificity (*p* > 0.05).

In a fourth experiment we evaluated the model on KGMU imagery with and without root-canal fillings or restorations (Fig. 3b). The model was trained exclusively on Charité data. We observed that the presence of root-canal fillings and restorations significantly improved the model performance compared in terms of both the sensitivity (31.7 ± 2.2% versus 16.5 ± 0.9%; *p* < 0.01) and the F1-score (42.7 ± 1.9% versus 25.8 ± 1.2%; *p* < 0.001), although no significant differences were detected for PPV and specificity, respectively (*p* > 0.05).

## Discussion

Generalizability and robustness of machine learning models are relevant properties not usually known a priori. Models which are not generalizable across populations or image sources will only be applicable in the setting they were developed in. A number of possible sources for limits in generalizability have been identified^3^. Generalizability of CNNs has not been explored in dentistry, while the number of research studies in the field is increasing rapidly^2^. We hypothesized that generalizability of deep learning models to detect apical lesions on panoramic images (as one exemplary use-case) was not given and that the models’ performance (measured via the F1-score) would significantly differ on test data from different centers. We confirm this hypothesis. Moreover, we showed that cross-center training can mitigate the lack of generalizability to some degree.

Our findings need to be discussed in more detail. First, we showed generalizability between the two evaluated populations is not given, as exhibited by a significantly lower sensitivity and F1-score. While we cannot ascertain if the same behavior also applies for other conditions and detection tasks, our findings are noteworthy for researchers in dental image analysis. Second, we found that population characteristics (including dental status) and image conditions differed between the two centers. Indian individuals had more teeth and fewer dental work experience (restorations, root-canal fillings), while images were brighter and had more contrast (as they stemmed from different x-ray machines but also as different exposure conditions may have been used). We started to explore the effects of image characteristics first, and tried to overcome the differences in the training dataset by data augmentation. However, this did not overcome the problem of lacking generalizability; obviously, image pixel value characteristics were not at the heart of the problem. This is relevant from two perspectives: (1) Data augmentation can only limitedly mitigate limits in generalizability, with other approaches being required. (2) Image characteristics differences may not be the main problem leading to limited generalizability, and researchers may want to explore heterogeneity of training data towards other aspects than image conditions. Third, we found that by training the models on an increasingly mixed dataset, adding more and more data from the second center (KGMU), generalizability improved. We showed that with increasing cross-center training, the sensitivity of the model for KGMU data increased, at only limited detriment for Charité data. Overall, adding KGMU data in a stepwise manner nearly mitigated the lack in generalizability. The increase in sensitivity (and F1-score) of the model on KGMU data was steepest when the first 20% of the Charité data were replaced but continued to increase further when replacing up to 100%. In fact, training only on KGMU data (100% replacement) led to the models nevertheless performing quite well on Charité data, which is noteworthy: Obviously, generalizability was not bidirectional in our experiments; models trained solely on KGMU data showed generalizability when applied to Charité data, but not vice versa. Fourth, we explored this behavior and concluded that differences in the dental status of the two populations were a key factor. The largest difference in model performance was identified when the models were tested on KGMU data with root-canal fillings or restorations being present and without any restorations being present. Models trained on Charité data generalized well on KGMU data with dental work experience, while the generalizability was poor on KGMU data without such experience. This might be, as in the (Charité) training data such work experience was quite common (indicating the differences in dental treatment provision), enhancing the model sensitivity for detecting apical lesions, possibly as the model exploits correlations between apical lesions and root-canal fillings or restorations. This may also explain that in our case the discussed generalizability was not bi-directional: Models trained on Charité data did not generalize well on KGMU data given the missing option to exploit this correlation, while models trained on KGMU data generalized better as they did not show this type of learning bias. Such a finding should encourage AI researchers to actively leverage clinical knowledge a priori, which may lead to better model performance as shown previously^9^. However, our findings should also raise the awareness of researchers, reviewers and practitioners that the complexity of dental radiographic imagery may yet be underrepresented in many studies and outcome metrics on a hold-out test which originates from the same population as the training set may yield overly optimistic estimates for the model’s generalizability.

This study has a number of strengths and limitations. First, and as a strength, it assessed generalizability, a highly relevant property of deep learning algorithms, and aimed to identify reasons for limited generalizability as well as how to overcome them. Our study will inform the definition of standards within the ITU/WHO Focus Group AI for Health (FG-AI4H). Second, and as limitation, it focused on one exemplary use-case, the detection of apical lesions on panoramic radiographs, while a large range of further pathological or non-pathological findings on the same imagery or other material (other radiographs, but also photos, scan data etc.) are of interest. Also, the image material stemmed from two centers, and generalizability may be more or less affected when considering further centers, but also further machinery etc. Hence, one cannot deduce that our findings will be applicable to other settings and challenges. Third, we performed only a limited range of experiments to understand and mitigate limitations in generalizability. It is noteworthy that it may well be that further parameters beyond root-canal fillings or restorations are similarly associated with the model’s performance. As a mean to overcome this difficulty, future studies could resort to apply methods of explainable AI to identify image level features and structures which are particularly relevant for the model. This could serve to identify correlation structures, contrast the areas of interest with those dentists use in their diagnostics performance, and safeguard the model against bias. Last, we used pixelwise metrics, which are easy to interpret and useful for this particular study. From a clinical perspective, it will be less important to identify the exact pixels, but the entities depicted by groups of pixels belonging to a same class. In previous studies, we used tooth-level metrics for this purpose^11^.

## Conclusion

In conclusion, deep learning models trained to detect apical lesions on panoramic radiographs did not necessarily show generalizability. Replacing training data from one center with data from the other center (cross-center training) improved the model performance. We identified the presence of dental work experience in the training dataset to significantly affect generalizability, while image characteristics (brightness, contrast) were less important. Researchers should aim to demonstrate generalizability of their models and should employ cross-center training to increase it. Understanding the reasons behind limits in generalizability will help to devise further strategies to mitigate generalizability problems. Clinicians should scrutinize deep learning applications for applicability in their setting of interest.

## Supplementary Information

Supplementary Information

## Data Availability

Data used in this study can be made available if needed within data protection regulation boundaries.
