# Supplementary material for: Generalizability of deep learning models for dental image analysis
Source: Sci Rep. 2021 Mar 17;11:6102. doi: 10.1038/s41598-021-85454-5 (PMC7969919; doi:10.1038/s41598-021-85454-5)
Supplement: Supplementary file 1 — Supplementary Information [file 41598_2021_85454_MOESM1_ESM.docx]

**Appendix**

**Generalizability of Deep Learning Models for Dental Image Analysis**

Joachim Krois^1*^, Anselmo Garcia Cantu^1*^, Akhilanand Chaurasia^2^, Ranjitkumar Patil^2^, Prabhat Kumar Chaudhari^3^, Robert Gaudin^4^, Sascha Gehrung^1^, Falk Schwendicke^1^,

^1^ Department of Oral Diagnostics, Digital Health and Health Services Research, Charité - Universitätsmedizin Berlin, Germany

^2^ Department of Oral Medicine and Radiology, King George’s Medical University, Lucknow, India

^3^ Division of Orthodontics & Dentofacial Deformities, AIIMS, New Delhi, India

^4^ Department of Oral and Maxillofacial Surgery, Charité - Universitätsmedizin Berlin

* joint first authors

**Corresponding author:**

Prof. Dr. Falk Schwendicke MDPH

Charité – Universitätsmedizin Berlin

Department of Oral Diagnostics, Digital Health and Health Services Research, Charité - Universitätsmedizin Berlin, Germany

Aßmannshauser Str. 4-6

14197 Berlin, Germany

Phone: 0049 30 450 62556

Fax: 0049 30 450 7562 556

falk.schwendicke@charite.de

*Evaluation metrics*

For every image *i*, the pixel-level confusion matrix elements were calculated: *true_positives_i* (the number of pixels correctly classified as apical lesion), *true-negatives_i* (the number of pixels not belonging to an apical lesion and correctly classified as such), *false-positives_i* (the number of pixels incorrectly classified by the model as apical lesions), *false_negatives_i* (the number of pixels belonging to apical lesion but missed by the model). The cumulative values of these elements were then calculated on the full set of images on the test set:

| $TP=\sum_{i} \text{true-positives}_{i}$ | $FP=\sum_{i} \text{false-positives}_{i}$ |
| --- | --- |
| $FN=\sum_{i} \text{false-negatives}_{i}$ | $TN=\sum_{i} \text{true-negatives}_{i}$ |

Based on these quantities, the following scores were computed:

$$Sensitivity=\frac{TP}{TP+FN}$$

$$PPV=\frac{TP}{TP+FP}$$

$$F1=\frac{2\times Sensitivity\times PPV}{Sensitivity+PPV}$$

$$Specificity=\frac{FN}{FN+FP}$$

*Data preparation and modeling*

All of the images and corresponding annotations were center-cropped inside the region of interest (ROI). The latter is defined by the minimal convex hull enclosing the jawbone image segment (Figure A1). The exclusion of non-relevant portions of the image has been recently shown to increase the predictive power of CNN models in dental classification tasks (Kim et al. 2019; Muramatsu et al. 2020). The cross-validation procedure is further detailed out in Figure A2.


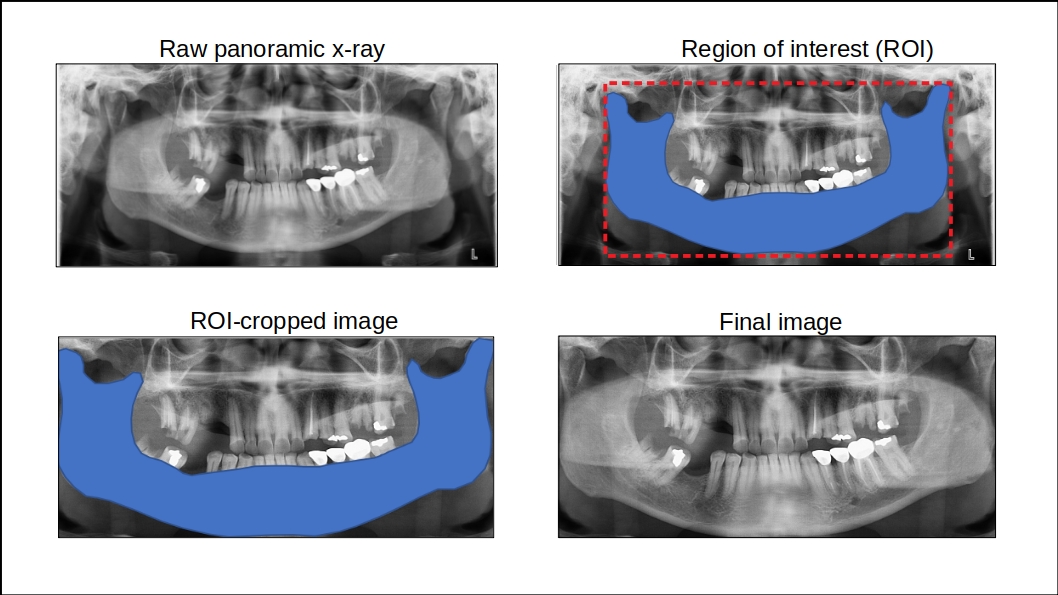

Figure A1. ROI cropping of panoramic x-ray scans. Color coding: jawbone segment (blue), the region of interest (red dashed line).


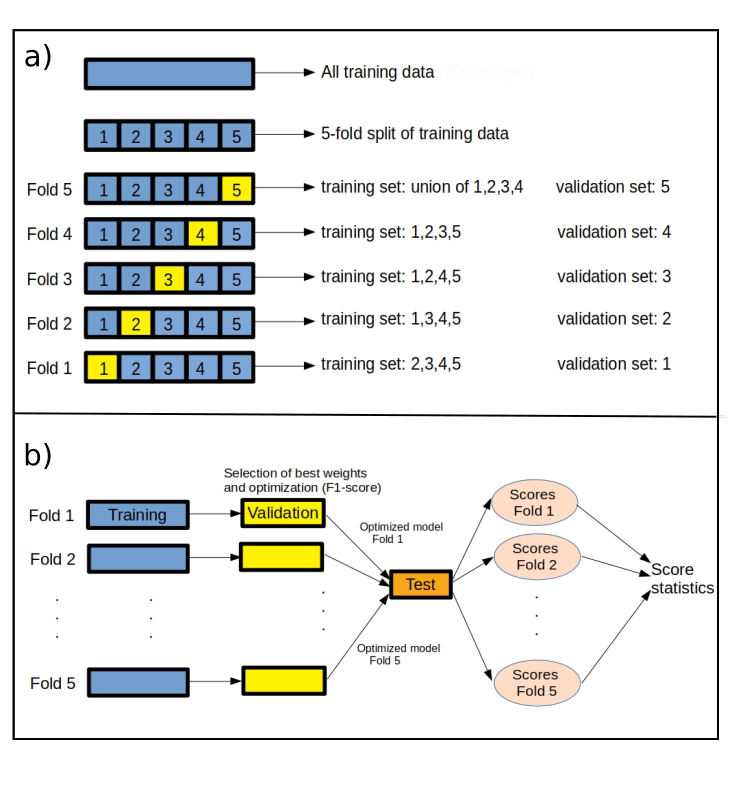


Figure A2. Illustration of the 5-fold cross-validation data preparation and training procedure: a) the general 5-fold approach; b) the use of the 5-fold approach for model training and evaluation.
